# Obesity Impact on SARS-CoV-2 Infection: Pros and Cons “Obesity Paradox”—A Systematic Review

**DOI:** 10.3390/jcm11133844

**Published:** 2022-07-02

**Authors:** Damiana-Maria Vulturar, Carmen-Bianca Crivii, Olga Hilda Orăsan, Emanuel Palade, Anca-Dana Buzoianu, Iulia Georgiana Zehan, Doina Adina Todea

**Affiliations:** 1 Department of Pneumology, Iuliu Hațieganu University of Medicine and Pharmacy, 400332 Cluj-Napoca, Romania; vulturar.damianamaria@elearn.umfcluj.ro (D.-M.V.); dtodea@umfcluj.ro (D.A.T.); 2 Morphological Sciences Department, Iuliu Hațieganu University of Medicine and Pharmacy, 400000 Cluj-Napoca, Romania; 3 5th Department Internal Medicine, 4th Medical Clinic, Iuliu Hațieganu University of Medicine and Pharmacy, 400015 Cluj-Napoca, Romania; olga.Orasan@umfcluj.ro; 4 Department of Cardiovascular and Thoracic Surgery, Iuliu Hațieganu University of Medicine and Pharmacy, 400332 Cluj-Napoca, Romania; emanuel.palade@umfcluj.ro; 5 Department of Pharmacology, Toxicology and Clinical Pharmacology, “Iuliu Hatieganu” University of Medicine and Pharmacy, 400337 Cluj-Napoca, Romania; abuzoianu@umfcluj.ro; 6 Department of Cardiology, Heart Institute, Iuliu Hațieganu University of Medicine and Pharmacy, 400001 Cluj-Napoca, Romania; zehan.iulia.georgiana@elearn.umfcluj.ro

**Keywords:** SARS-CoV-2 infection, COVID-19, “obesity-paradox”, intensive care unit, mortality

## Abstract

Background: During the last years, the COVID-19 pandemic meets the pandemic generated by obesity, raising many questions regarding the outcomes of those with severe forms of infection. Methods: The present systematic review summarises and analyses the data providing evidence for or against the “obesity-paradox” in COVID-19 patients. After applying the inclusion and exclusion criteria, 23 studies were included. We also analysed the presumably underlying basic mechanisms. Results: The patients with a body mass index (BMI) of 30–40 kg/m^2^ presented severe symptoms that led to intensive care unit (ICU) admission but not increased death rate. Those with a higher degree of obesity, with a BMI higher than 40 kg/m^2^, led to a rise in the death rate, particularly in young patients. Obesity was associated with a higher rate of ICU admission but was not determined as an independent predictor of increased mortality. In contrast, some studies suggest a strong association between obesity or morbid obesity and the risk of death. Conclusions: The existence of “obesity-paradox” cannot be stated; our study presents obesity as a critical risk factor in the evolution of COVID-19.

## 1. Introduction

The coronavirus pandemic burst in December 2019, with the first case declared in Wuhan, China, and transcended the borders, reaching the whole world. It has quickly become an unprecedented worldwide crisis in the public health sector.

The disease results from coronavirus infection, subsequently named severe acute respiratory syndrome coronavirus 2 (SARS-CoV-2). International Committee on Taxonomy of Viruses (ICTV) announced “severe acute respiratory syndrome coronavirus 2 (SARS-CoV-2)” as the name of the new virus on 11 February 2020. This name was chosen because the virus is genetically related to the coronavirus responsible for the SARS outbreak of 2003. While related, the two viruses are different [1]. Previously named 2019 novel coronavirus (2019-nCoV), or human coronavirus 2019 (HCoV-19 or hCoV-19), SARS-CoV-2, a member of Sarbecovirus, beta-CoV lineage, is a positive-sense single-stranded RNA (+ssRNA) virus with a nucleocapsid protein (N) that holds the RNA genome and a viral envelope formed by three proteins—spike (S), envelope (E) and membrane (M). The S-proteins are glycoproteins type I membrane with a single transmembrane domain-oriented on the extracellular side [2,3,4,5].

The spike protein contains a receptor-binding domain that recognises the angiotensin-converting enzyme 2 (ACE2) receptor, binding to it. After the fusion with the ACE2 receptor, the viral genome is released into the cytoplasm. The viral mechanisms of replication are activated, leading to the uncontrolled multiplication of the viral load [6,7]. The distribution of the ACE2 receptor throughout different tissues explains the clinical presentation of COVID-19 disease, with the predominance of respiratory symptoms: shortness of breath, dry cough, chest pain and sore throat; other non-specific symptoms are fever, tiredness, headache, loss of smell or taste and diarrhoea [8].

Some conditions such as age, male gender, cardiovascular disease, chronic lung disease, kidney disease, malignancy, hypertension, diabetes and obesity are associated with developing a more severe form of the disease with the need for hospitalisation [9,10].

Overweight and obese are characterised by an abnormal or excessive fat accumulation that raises a health risk [11]. Being obese leads to cardiovascular disease, type 2 diabetes, hypertension, coronary heart disease and some cancers. These conditions are associated with a high risk of mortality in COVID-19 [12,13,14]. A direct relationship between the class of obesity and worse outcomes was shown in patients infected with SARS-CoV-2 [15]. However, the results concerning the association between BMI and hospital mortality, especially in ICU units, are conflicting. Some studies reported that obesity does not interfere with survival rate, whereas others showed an association between obesity and death. Ironically this is named the “obesity paradox”, and it was also seen in cardiovascular disease (heart failure), end-stage renal disease, respiratory disease, including chronic obstructive pulmonary disease, pulmonary embolism and acute respiratory distress syndrome [16,17,18,19]. This paradox means that obesity confers a survival advantage in clinical subpopulations, being protective and associated with a decreased mortality rate.

The nutritional status of the patients is evaluated using the BMI obtained by dividing the body weight (kilograms) by the squared height (m^2^). It is used to classify overweight and the classes of obesity (class I, II, III) in clinical practice. According to World Health Organization data from 2016, the percentage of people with increased BMI has reached 39%, and it is now considered a global health issue [20,21]. The existing literature emphasises that the prevalence of obesity is around 42.4% in the American population, 19.5% in Brazilians and 10–30% in Europeans [22,23,24]. Interestingly, WHO proposed different cut-off values for overweight and obesity in the Asian population because of their different biotype [25,26]. The problem in China is that the obesity rate is growing by around 5–6% in the areas where fast food is favoured, which has reached more than 20% [27].

BMI marker cannot distinguish between the distribution of subcutaneous or visceral adipose tissue. It is known that excessive visceral fat is associated with systemic inflammation. Moreover, the expression of ACE2 receptors is higher in the visceral fat tissue than in subcutaneous fat, which hosts a greater quantity of viral load, resulting in more severe forms of the disease in patients with much visceral fat. This group of patients is more likely to need admission to the intensive care unit (ICU), requiring invasive mechanical ventilation and presenting an increased risk of death [28,29].

In critically ill patients infected with SARS-CoV-2, the relationship between obesity and mortality is not clear. The association between obesity and ICU admission/mortality is contradictory, and there is no established conclusion concerning the effects of excess weight on COVID-19 evolution. The “obesity-paradox” theory is debatable, so this systematic review aims to determine the influence of obesity in the evolution of patients with COVID-19, evaluate the impact of high BMI on ICU admission and in-hospital mortality and test the “obesity-paradox” theory.

## 2. Methods

### 2.1. Literature Search

The Preferred Reporting Items were used for Systematic Reviews and Meta-Analyses, known as PRISMA [30]. The articles were selected from different databases by using PubMed, Research Gate and Web of Science using the following keywords: COVID-19, SARS-CoV-2 infection, obesity, intensive care unit and mortality. Additionally, a manual search was carried out.

The research was carefully realised using the following keywords: ((((((COVID-19) OR (SARS-CoV-2)) OR (2019 novel coronavirus)) OR (severe acute respiratory syndrome coronavirus 2)) AND (obesity)) AND (intensive care unit)) AND (mortality). Selected research papers ranged from January 2020 to December 2021. After the initial search through databases, we conducted also a manual search and we found 4 studies eligible to be included in the analysis.

### 2.2. Inclusion Criteria

Inclusion criteria were as follows:

1. Studies published between January 2020 and December 2021
2. Studies published in English
3. Studies measuring obesity by BMI
4. Studies that must have been longitudinal studies (retrospective or prospective or clinical trial)
5. Studies that examined the association between obesity and mortality, specifically in SARS-CoV-2 infection

Exclusion criteria were as follows:

1. Studies published as abstracts, letters to the editor, book chapters
2. Studies regarding animal model
3. Systematic reviews and meta-analysis
4. Studies on paediatric patients or pregnant women.

## 3. Results

**Figure 1:**
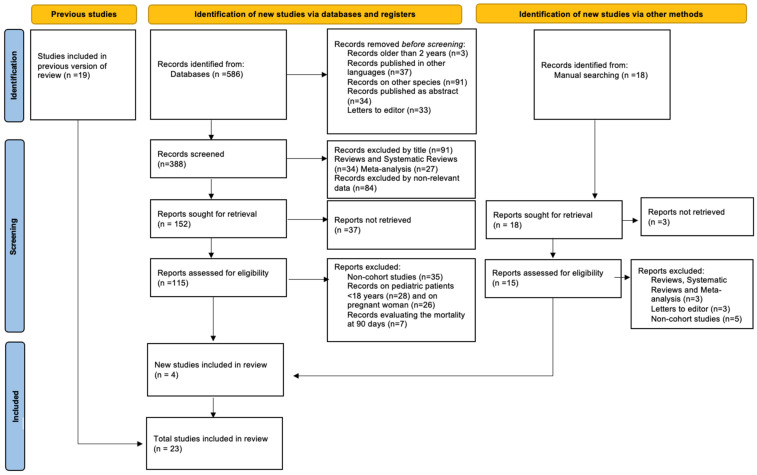
Flow diagram of literature search, screening, inclusion and exclusion of studies.

From the proposed review, a total of 586 articles were identified. Inclusion and exclusion criteria were applied to select only the relevant papers for this systematic review (Figure 1).

Nineteen studies were included in the initial selection, and the information from all these is represented in Table 1 and Table 2. After conducting the manual search, we found another 4 articles suitable for our review. Finally, we included 23 articles. Of the included studies, 12 reported mortality rates, 3 reported ICU admission and 8 reported ICU admission and mortality. Eleven studies originated from the USA, three originated from France, three originated from Italy, one originated from Europe (including Italy, Spain and Germany), one originated from the UK, one originated from Mexico, one originated from Brasilia and one originated from Morocco. The geographical distribution of these studies is relevant for a global analysis of the impact of obesity on the evolution of COVID-19. Diagnosis of COVID-19 and definitions of obesity in the included studies are represented in Table 1 and Table 2.

The positive diagnostic of the infection with SARS-CoV-2 was confirmed by detecting the viral RNA from respiratory samples determined by specific real-time reverse transcriptase-polymerase chain reaction (RT-PCR).

Of the 23 selected articles, 10 support the “obesity-paradox” theory (Table 1), while 13 articles highlight the correlation between obesity and the unfavourable evolution of SARS-CoV-2 infection (Table 2).

Studies confirming “obesity-paradox” have found that the patients with a BMI from 30–40 kg/m^2^ presented severe symptoms that led to ICU admission but not to an increase in the death rate. The patients with a higher degree of obesity, with a BMI higher than 40 kg/m^2^, led to a rise in the death rate, particularly in young patients. Obesity was associated with a higher rate of ICU admission but was not determined as an independent predictor of increased mortality. There are also studies suggesting a strong association between obesity or morbid obesity and the risk of death.

## 4. Discussion

The impact of obesity on patients with COVID-19 infection has been debated since the beginning of the pandemic. Studies from the onset of this global public health challenge have presented the negative effect of obesity on COVID-19 disease. Over time, the findings have been more nuanced, leading to studies that did not identify significant differences in mortality between obese and non-obese patients infected with COVID-19. The role of obesity in COVID-19 infection became unclear. Ironically, despite the increased health risk associated with obesity, some studies suggest better outcomes for obese hospitalised patients, including mortality, a phenomenon referred to as the “obesity paradox”.

The studies supporting the theory concluded that obesity was not an independent risk factor for survival rate or hospitalisation length [34], even for mortality rate [31,33,53]. There are data underlying the idea of higher mortality in normal weighted patients than in overweight or obese patients, the explanation being held by age differences [32]. On the other hand, there are studies supporting the idea that obesity is correlated only with the risk of ICU admission but not with death. A likely answer is that obesity prevalence decreases with age [38]. Obesity is linked with increased odds of death in Black patients but not in the non-Black subgroup. Nonetheless, obesity was independently associated with the need for ICU admission and invasive mechanical ventilation in the overall population [37]. After the initialisation of mechanical ventilation, the odds of death are similar for all patients, despite the differences in their BMI. Moreover, the underweight group presented an increased risk of death, whilst no association with the need for intensive mechanical ventilation was found [35,36]. In the evolution of the disease, those with mild and moderate obesity are predisposed to develop more severe ARDS, and paradoxically they recover better [39].

A higher risk of medical intensive care need and death is found in patients with three or more underlying comorbidities [38]. There are parameters, such as age, chronic cardiac and pulmonary diseases and elevated IL-6 and D-dimers, associated with the unfavourable evolution of COVID-19 [31]. Additionally, male sex, immunosuppression and inflammatory markers such as C-reactive protein, neutrophilia and lymphopenia are associated with the severity of the disease and death [53].

In antithesis, the studies suggesting a strong association between obesity or morbid obesity and the risk of death noticed that obesity is an aggravation factor of the evolution of COVID-19. The longer lengths of hospitalisation, the need for intubation and the high mortality rate are associated with obesity [40,43,45,46,47].

Studies have shown primary outcomes, such as mortality, respiratory failure and sepsis and secondary effects, such as the need for oxygen through the nasal cannula, non-invasive ventilation and invasive ventilation. Clinically, the patients have had a cardiac failure, haemorrhage and different types of embolism. The prone position and extracorporeal membrane oxygenation (ECMO) were substantially more necessary in overweight and obese patients than in normal-weight individuals [45]. The association between BMI, ventilation management and clinical outcomes in patients with ARDS and COVID-19 mentions a higher risk of 28-day mortality only in patients with severe ARDS and class II obesity [46].

A high risk of death was found in males and younger patients with increased BMI [41], with a statistically significant association between the obesity prevalence and the mortality rate [48]. In addition, Bello-Chavolla et al. showed that obesity increased the risk of mortality [42], and the rate of ICU admission is directly proportional to the BMI class [44,50]. It was shown that obesity through its association with systemic inflammation with high levels of C-reactive protein is a mediator of fatal outcomes in SARS-CoV-2 infection [49]. Moreover, obesity and its related conditions lead to severe outcomes [51]. Interestingly, another study showed the combination of slow-walking pace with obesity in the development of severe forms of infection with higher rates of mortality [52].

### 4.1. Severe COVID-19 in Higher Obesity

Obesity has become a worldwide epidemic linked to various health problems and chronic diseases. Different metabolic pathologies (including type 2 diabetes and dyslipidaemia), hypertension, cardiovascular and lung diseases, cancer and obstructive sleep apnoea have been associated with obesity, which is considered a risk factor. These diseases are commonly reported as comorbidities in severe forms of COVID-19 [54,55,56,57,58,59].

A study of SARS-CoV-2 infection from 50 countries, using worldwide epidemiological data, emphasised the prevalence of obesity/overweight and mean BMI, which were significantly correlated with the disease [60].

Obesity-related pathologies are characterised by metabolic syndrome, which is correlated with an increased risk of COVID-19. A population-based study performed in an extensive commercial database (Explorys IBM) with electronic health records from 26 national healthcare systems, including patients with COVID-19, between December 2019 and May 2020, found a direct relationship between metabolic syndrome components and the risk of COVID-19. The increased cumulative prevalence of COVID-19 in those suffering from a metabolic syndrome was evident [odds ratio (OR), 7.00; 95% confidence interval (CI), 6.11–8.01]. The patients with hypertension, obesity, diabetes mellitus and dyslipidaemia were found with higher morbidity and mortality in COVID-19 [61].

In a study of the patients included in the UK Biobank (UKBB), Scalsky et al. analysed the BMI, diabetes, dyslipidaemia and SARS-CoV-2 infection. The study considered patients from 16 March to 29 June 2020. Higher BMI, type 2 diabetes and haemoglobin A1c (HbA1c) were associated with an increased risk for SARS-CoV-2 infection. In contrast, elevated high-density lipoprotein (HDL) and apolipoprotein A and alcohol intake (red wine) was associated with decreased risk of COVID-19. The study concluded that high HDL levels protect against SARS-CoV-2 [62]. As well, dyslipidaemia is correlated with severe COVID-19 (relative risk (RR), 1.39; 95% CI, 1.03–1.87; *p* = 0.03). Hariyanto et al. reported the results after a meta-analysis of all PubMed articles presenting data on dyslipidaemia and COVID-19. Obesity and metabolic syndrome with dyslipidaemia are aggravating factors of COVID-19 infection [63].

An interesting study evaluated the association of cardiometabolic traits with COVID-19 susceptibility. Genetic factors implicated in increased BMI were causally associated with testing positive for COVID-19 (OR, 1.08; 95% CI, 1.03–1.13). Genetic evidence supported BMI as a causal risk factor for COVID-19 susceptibility [64].

A meta-analysis of the correlation between obesity and COVID-19 indicated that the disease was more severe in COVID-19-positive obese patients than in non-obese patients [12].

Over time, a better outcome was observed in obese patients infected with the influenza virus. This phenomenon refers to the “obesity-paradox” [65]. In addition, the “obesity-paradox” was revealed in different pathological entities, such as end-stage kidney disease, COPD, pulmonary embolism and other cardiovascular pathological conditions [17,66,67]. Nowadays, this paradox can be discussed (a higher BMI associated with lower mortality, more extended hospitalisation, ventilation and ICU requiring mechanical ventilation) in the development of severe forms of COVID-19 pneumonia because the pathogenesis of severe COVID-19 related to obesity is still unclear.

Lavie et al. showed in their paper that there is an obesity paradox in COVID-19, the mortality being lower in obese patients than in normal-weight ones. In the overweight patients, the mortality was 36% lower than in the normal-weight group, while in obese patients, the mortality rate was 45% lower than in normal-weight individuals. Moreover, they claim that underweight patients present the highest 90-day mortality rate. This phenomenon is expectable considering the comorbidities such as severe chronic obstructive pulmonary disease or cancer [16]. Furthermore, Arbel et al. have realised a study to examine the potential association between SARS-CoV-2 infection and obesity at a state-wide level in the USA using a mathematical model. The results of their study support the theory of the obesity survival paradox [68].

Several hypotheses are trying to explain this phenomenon. On the one hand, it could be the case of a wrong ARDS diagnosis instead of the actual condition (referring to atelectasis resulting from the elevated pleural pressures associated with rigid chest walls). On imaging, this could be confounded with lung infiltrates [69]. On the other hand, another hypothesis claims that it is plausible that minor lung injury in obese patients has resulted from these patients receiving smaller amounts of fluid resuscitation [70]. A third hypothesis says that in obese patients, the proinflammatory background provides resistance to critical illness [71]. Another theory refers to the decreased rate of iatrogenesis in obese patients due to fewer manoeuvres in the ICU [47].

**Figure 2:**
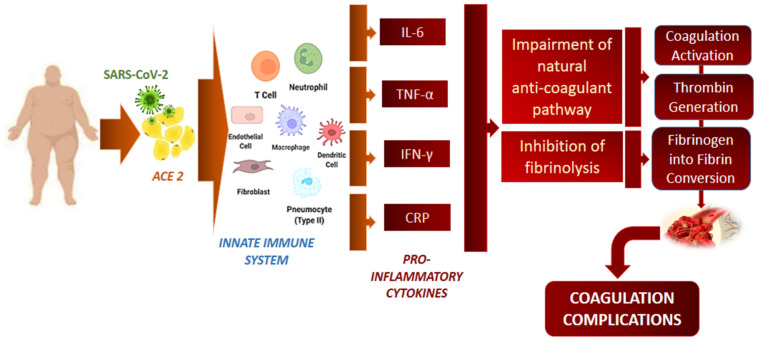
**The relation between SARS-CoV-2 infection and coagulation complications.** Infection with SARS-CoV-2 stimulates the innate immune system by releasing proinflammatory cytokines. Interleukin (Il)-6, tumour necrosis factor (TNF)-α, interferon (IFN)-γ and C-reactive protein (CRP) interfere with the anticoagulant pathway and inhibit fibrinolysis. Due to all these mechanisms, coagulation is stimulated and ends with fibrin development.

It cannot be excluded that obesity is associated with a more severe ARDS, obese patients having an impaired haemostatic balance leading to thrombosis [72,73] (Figure 2). The use of anticoagulants in obese patients is controversial, with limited guidelines regarding the treatment decision. The emergence in obese COVID-19 patients is the thromboembolic complications [74]. The lack of anticoagulant use at the beginning of the pandemic is thought to have led to a high mortality rate, and obesity cannot be considered an independent risk factor for mortality. In this context, Drakos et al. showed that obese patients (with a BMI over 30) treated with early aggressive anticoagulants with enoxaparin or heparin had a half-death rate than those who received basic thromboprophylaxis (26% vs. 61% mortality, respectively, *p* = 0.000). The same study demonstrated that obesity was not associated with higher mortality rates for critically ill intubated patients than non-obese patients. [75]. All these data reflect the importance of treatment in the evolution of patients with COVID-19, noting remarkable differences between studies conducted at the beginning of the pandemic and later studies.

On the other hand, obesity reduces the chest-wall elastance, with lower respiratory system compliance and a consequent reduction of the expiratory reserve volume, increasing the risk of infections. Furthermore, obesity affects the total lung capacity and the process of ventilation-perfusion. However, in the pathogenesis of acute respiratory distress syndrome (ARDS) from COVID-19, this pre-existent inflammatory environment is thought to offer a protective resistance to the high influx of inflammatory cytokines, known as a “cytokine storm” [76]. As part of the ARDS management, the prone position, by preventing small airway closure preceding end-expiration, improves the kinetics of inspiration, reducing mortality and benefiting the outcomes [77,78]. When turning the patients from supine to prone position, the alveolar volume improves more in patients with BMI > 30 kg/m^2^ than those with BMI less than 25 kg/m^2^.

Studies highlighting the protective role of obesity in critically ill patients concluded that other factors than BMI might explain the severity of pneumonia with ARDS. The relationship between epicardial and visceral obesity should also be considered [32]. Obesity definition also includes visceral fat, perivascular fat and epicardial adipose tissue. In this framework, the critically ill patients with ARDS, presenting visceral adiposity, increased the mortality rate [79]. Furthermore, visceral adipose tissue has been associated with more severe COVID-19 than total BMI [80,81].

In a cohort study of 192 patients infected with SARS-CoV-2, Conte et al. found that obesity, as an independent predictor, was not associated with the severity of the disease. Furthermore, they showed that with systemic inflammation (C-reactive protein), PaO2/FiO2 and hyperglycaemia, epicardial adipose tissue inflammation was significantly associated with ICU hospitalisation, invasive ventilation and death [82].

Another theory supporting the favourable prognosis of obese patients is that high energy stores could counteract catabolic stress and energy-consuming situations caused by severe ARDS infections [18], especially SARS-CoV-2 infection [82]. Furthermore, this excessive fat accumulation offers increased metabolic reserves that can withstand the drop in calorie intake that usually occurs when patients are admitted to ICU with sepsis or lung injuries induced by mechanical ventilation [34].

It is known that cytokine release syndrome (CRS) is associated with severe disease. Furthermore, it was pointed out that adipocytes can continuously release proinflammatory cytokines. In this way, obesity is related to a low level of chronic inflammation. Given this regular proinflammatory background, the acute phase of the immune response leads to a more significant elevation of the CRP level (Figure 2) [83]. However, some studies have not found a significant difference between CRP levels in obese and non-obese patients [34].

A study by Stapleton et al. on 1409 patients with acute respiratory distress syndrome demonstrated that those patients with obesity and ARDS have lower biomarkers of inflammation (IL-6 and IL-8 cytokines) and surfactant protein D (a marker of alveolar epithelial injury) than those with normal weight. Furthermore, the same study found no association between obese patients with ARDS and mortality rate [84]. The levels of these cytokines in healthy and obese patients are elevated [85,86]. In obese patients, the innate and inflammatory responses may be attenuated during ARDS, reducing alveolar epithelial injury.

### 4.2. The Role of ACE2 Receptors in COVID 19 Infection

Increased circulating ACE2 levels were observed in smokers, diabetics and obese patients [87,88]. In obesity, the expression of ACE2 is enhanced, leading to viral entry into the cells via ACE2 receptors. The number of ACE2 receptors in adipose tissue is higher than in the lungs, suggesting that adipose tissue is more prone to infection. In obese patients, who have a higher amount of adipose tissue, the level of ACE2 receptors will also be higher. In these patients, in case of a COVID-19 infection, the adipose tissue becomes a viral reservoir due to the increased number of ACE2 receptors that allow a more significant amount of virus to enter the cells [88,89,90].

The variable expression of ACE2 in different tissues such as the heart, kidneys and gastrointestinal tract tests may explain the susceptibility to the infection, the symptom manifestations and the outcomes of the SARS-CoV-2 infection. The ACE2 distribution in the upper part of the oesophagus and the enterocytes from the ileum and colon can explain gastrointestinal symptoms. They can lead to the possibility of a faecal–oral transmission route. Due to the high expression of ACE2 in the heart tissue, those with cardiovascular pathologies (ischemic heart failure) are more susceptible to getting infected and progressing to critical disease [91,92].

Interestingly, there is a study analysing the cell types included in the adipose tissue and which of them are responsible for the expression of ACE2. The cell types included in this analysis were adipocytes, microvascular endothelial cells and macrophages. They found that ACE2 was higher expressed in the microvascular endothelial cells and lower expressed in the macrophages. The expression of ACE2 was not associated with the adipocyte’s subpopulation. This fact can explain that the severity of COVID-19 in obese patients is given by the microvascular damage and the increased risk of thrombotic events [89]. Furthermore, the same study showed that lower adipose tissue of ACE2 was associated with diabetes, obesity status, BMI and increased insulin and triglyceride levels [93].

### 4.3. The Implication of Vitamin D in COVID-19 Obese Patients

**Figure 3:**
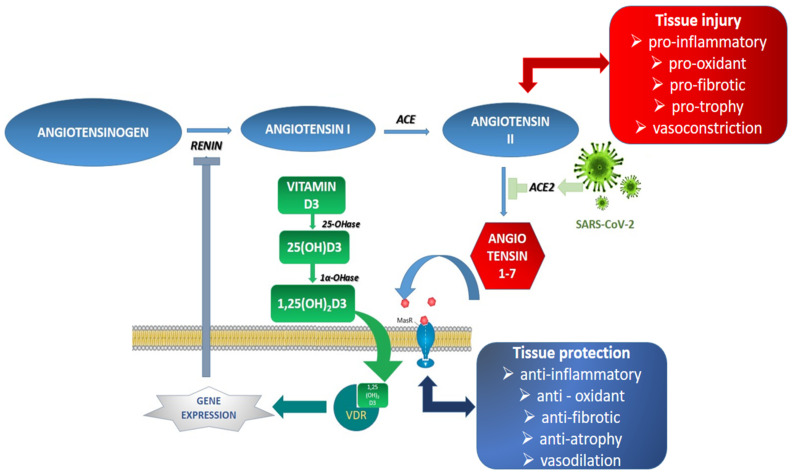
**Renin–angiotensin system: SARS-CoV-2 infection and implication of vitamin D.** In SARS-CoV-2 infection, virus attachment to the ACE2 receptors blocks angiotensin II conversion into angiotensin 1,7 and leads to tissue injury reactions. Vitamin D function cascade [vitamin D3 is produced in the skin under ultraviolet radiation exposure, 25(OH)D3—25-hydroxyvitamin D (calcifediol, ergocalciferol), 1,25[OH]_2_D3—1,25-dihydroxy vitamin D (calcitriol)] and vitamin D receptor (VDR) are implicated in the suppression of the renin gene expression, thereby inhibiting the renin-angiotensin system. Renin catalyses the conversion of angiotensinogen to angiotensin I, which is further converted to angiotensin II. Normally, angiotensin II turns into angiotensin 1–7—the tissue protector via MasR receptors.

Vitamin D plays an essential role in COVID-19 pathogenesis related to obesity. It was established that low levels of 25-hydroxyvitamin D have been associated with COVID-19 severity and mortality [94]. It is known that obese people have lower vitamin D levels. This molecule is essential in the proper functioning of the immune system by modulating the main proinflammatory cytokine (IL-6, TNF-alpha and interferon-gamma), managing the response mediated by Th1 lymphocytes and modulating the coagulation pathways. Another possibility is that vitamin D could decrease viral replication through a process mediated by some antimicrobial defence proteins (cathelicidins, defensins and IL-37) [95]. Moreover, vitamin D inhibits renin expression and generation as a negative renin–angiotensin system (RAS) modulator. This increases the ACE2/angiotensin 1-7/MasR axis activity, producing a potential protective role against acute tissue injuries [96,97] (Figure 3).

Campi et al. showed in a study that the levels of 25(OH)D (25-hydroxyvitamin-D, the active metabolite of vitamin D) were lower in those hospitalised with severely symptomatic disease than in those with mild disease or in non-SARS-CoV-2-infected controls. Furthermore, the same study revealed that the levels of 25(OH)D are a good indicator for predicting the severity of the disease, ICU admission and mortality in those severely symptomatic [96].

The present study reveals that the evolution of patients with COVID-19 is different depending on the received treatment. In time, this has adapted to the understanding of the mechanisms of viral action. In conclusion, obesity, with all the manifestations generated by COVID-19, cannot be considered a plus in the evolution of the disease. It is obvious that a high degree of obesity leads to an increase in the rate of ICU admission and mortality. On the other hand, the immune status of patients is also important. In general, the studies in question do not refer to the immune status of patients. Of course, patients with comorbidities are more susceptible, they have different degrees of immune impairment, with unfavourable evolution. In addition, supplements with vitamin D, a stimulant of immunity, have been auspicious in the evolution of patients; it further emphasises the role of immunity in the evolution of COVID-19. All these data lead us to the fact that a real “obesity paradox” cannot be sustained.

### 4.4. Limitations and Strengths of the Study

At the same time, the limitations are the strengths of this study. The geographical distribution of the studies under discussion leads to a global picture of the impact of obesity on the COVID-19 evolution, with information for different categories of patients. However, the limitations are significant and decisive for the results and their interpretation. So, ethnic race, gender and age difference are essential for results. Furthermore, there is no adjustment for comorbidities, history of smoking, or socio-economic variables that can interfere with the disease severity and outcomes. It should be marked that patients presented to the hospital at different times after symptoms onset so that it can affect the clinical course. In addition, another significant limitation is the treatment of patients, knowing that it was different at the beginning from the one used after 6–8 months of pandemic evolution. In addition, also the biomarkers associated with the disease severity are not the same in all the studies. The values of the BMI to define obesity in the included studies are also different, with the mention that the weight measurements could be inaccurate in the ICU units. The variety of healthcare systems, with different possibilities of access to hospitals, multiple testing strategies and diverse indications for hospitalisation and ICU admission, are other limitations. Another limitation is the lack of detailed ventilation information regarding ventilation pressures and the lack of information on the distribution of excess weight that can interfere with pleural pressures and ventilatory mechanics.

## 5. Conclusions

This review analysed the pros and cons of the “obesity paradox” and concluded that the theory is not plausible in obese patients with severe forms of COVID-19 infection. It can be highlighted the fact that those with obesity due to their immune system, and their obesity-related comorbidities are more susceptible to developing critical illness, with higher rates of ICU admission, invasive mechanical ventilation and death. In the ICU, obese patients with serious infections should be treated more aggressively for a better evolution and prognosis. However, this study encourages further scientific efforts to analyse the impact of obesity on COVID-19 pathophysiology.

The message that should be understood worldwide is that lifestyle interventions should be applied to lessen the risk in future waves of this viral pandemic. Today, the healthcare system needs to approach two different pandemics: obesity and COVID-19, and both can be tackled by filling the “prevention gap”.

## Figures and Tables

**Table 1:** Studies supporting the theory of “obesity-paradox”.

No.	Authors	Study design No. of Participants Country	Diagnosis of COVID-19	Measure of Obesity	Outcomes	Results/Conclusions
1.	Cummings et al. [31]	Prospective observational cohort study (*n* = 257) in New York-USA	RT-PCR	Obesity (BMI > 30 kg/m^2^) Severe obesity (BMI > 40 kg/m^2^)	Mortality-in hospital	The results of the study did not identify morbid obesity with a BMI ≥ 40 as an independent risk factor for mortality in COVID-19 disease.
2.	Busetto et al. [32]	Retrospective cohort study (*n* = 92) in Italy	RT-PCR	Normal weight (<25 kg/m^2^) Overweight (from 25 to <30 kg/m^2^) Obesity (≥30 kg/m^2^)	ICU admission IMV	A protective effect of obesity (obesity paradox) or other factors not related to BMI can explain the lack of worsening of the severity of the disease.
3.	Goyal et al. [33]	Retrospective cohort study (*n* = 1687) in New York- USA	RT-PCR	Underweight (<18.5 kg/m^2^) Normal (18.5 to 24.9 kg/m^2^) Overweight (25.0 to 29.9 kg/m^2^) Mild to moderate obesity (30.0 to 39.9 kg/m^2^) Morbid obesity (≥40.0 kg/m^2^)	In-hospital mortality	The study concluded that obesity was not an independent risk factor for in-hospital mortality, providing insights regarding a plausible obesity paradox in COVID-19.
4.	Biscarini et al. [34]	Retrospective cohort study (*n* = 427) in Italy	RT-PCR	Obesity (BMI ≥ 30 kg/m^2^)	ICU admission Mortality in ICU Mortality	The obesity does not interfere with survival rate or hospitalization length.
5.	Dana et al. [35]	Prospective study (*n* = 226) in France	RT-PCR	Underweight (<18.5 kg/m^2^) Normal (18.5 to 24.9 kg/m^2^) Overweight (25.0 to 29.9 kg/m^2^) Mild to moderate obesity (30.0 to 39.9 kg/m^2^) Morbid obesity (≥40.0 kg/m^2^)	In-hospital mortality	Interestingly, the mortality rate was lower in those with moderate obesity and overweight compared to those with normal weight and severe obesity, challenging the paradox of obesity.
6.	Kaeuffer et al. [32]	Prospective study (*n* = 1045) in France	RT-PCR	Normal weight (<25 kg/m^2^) Overweight (from 25 to <30 kg/m^2^) Obesity (≥30 kg/m^2^)	In-hospital morality	It has been demonstrated that the factors associated with an increased risk of death were the age, male sex, and immunosuppression and not the obesity.
7.	Kim et al. [36]	Retrospective study (*n* = 10,861) in New York USA	RT-PCR	Underweight (<18.5 kg/m^2^) normal weight (18.5 to 24.9 kg/m^2^) Overweight (25.0 to 29.9 kg/m^2^) Class I (30.0 to 34.9 kg/m^2^) Class II (35 to 39.9 kg/m^2^) Class III (≥40.0 kg/m^2^)	IMV In-hospital morality	Once intubated there are no statistical differences in death rate between obese patients and normal weight individuals.
8.	Yoshida et al. [37]	Retrospective study (*n* = 776) in New Orleans-USA	RT-PCR	Morbid obesity (BMI ≥ 40 kg/m^2^)	ICU admission IMV Mortality	No association between obesity and death was found in the non-Black group of patients.
9.	Kim et al. [38]	Multi-site, geographically retrospective study (*n* = 2491) in USA	RT-PCR	Obesity BMI ≥ 30 kg/m^2^ Severe obesity BMI ≥ 40 kg/m^2^	ICU admission In-hospital morality	Despite the higher prevalent of obesity in the study, there was found only an increased risk for ICU admission, but not for death.
10.	Mankowski et al. [39]	Retrospective study (*n* = 309) in New Orleans-USA	RT-PCR	Obesity (BMI ≥ 30 kg/m^2^)	IMV In-Hospital Mortality	Even though obese patients required more invasive mechanical ventilation, there was no difference in risk of in-hospital mortality.

**Table 2:** Studies not supporting the theory of “obesity-paradox”.

No.	Authors	Study Design No. of Participants Country	Diagnosis of COVID-19	Measure of Obesity	Outcomes	Results/Conclusions
1.	Halasz et al. [40]	Retrospective cohort study (*n* = 242) in Italy	RT-PCR	Underweight (<18.5 kg/m^2^) Normal weight (18.5–25 kg/m^2^) Overweight (25–30 kg/m^2^) Obese class I (30–35 kg/m^2^) Obese class II (35–40 kg/m^2^) Obese class III (>40 kg/m^2^)	Mortality	Severe obesity is associated with a greater mortality rate in individuals that were invasively ventilated, the study not being able to validate the theory of the obesity paradox.
2.	Tartof et al. [41]	Retrospective cohort study (*n* = 6916) in California	RT-PCR	Underweight (less than 18.5 kg/m^2^) Normal (18.5 to 24 kg/m^2^) Overweight (25 to 29 kg/m^2^) Obese class I (30 to 34 kg/m^2^) Obese class II (35 to 39 kg/m^2^) Obese class III or extreme obesity (>40 kg/m^2^)	In-hospital morality	There is a relationship between BMI and death, as BMI increases, the risk for death also increases, with more than 4 times for the highest BMI.
3.	Bello-Chavolla et al. [42]	Retrospective study (*n* = 51,633) in Mexico	RT-PCR	N/A	In-hospital morality	Obesity increases the risk of bad outcomes in COVID-19 disease, including mortality.
4.	Arjun et al. [43]	Retrospective study (*n* = 142) in USA	RT-PCR	Nonobese (BMI < 30 kg/m^2^) Obese (BMI >30 kg/m^2^)	ICU admission Mortality-in hospital	The study did not support the theory of obesity paradox in COVID-19.
5.	Czernichow et al. [44]	Prospective study (*n* = 5795) in France	RT-PCR	Underweight (<18.5 kg/m^2^) Normal weight (18.5–25 kg/m^2^) Overweight (25–30 kg/m^2^) Obese class I (30–35 kg/m^2^) Obese class II (35–40 kg/m^2^) Obese class III (>40 kg/m^2^)	Mortality	The study showed that mortality rate was higher in those with obesity.
6.	Abumayyaleh et al. [45]	Retrospective cohort study (*n* = 3635)	RT-PCR	Obese patients (BMI > 30 kg/m^2^)	In-hospital morality	It was pointed out the absence of evidence for the obesity paradox in COVID-19 patients.
7.	Schavemaker et al. [46]	Prospective study (*n* = 1122) Netherland	RT-PCR	Normal weight (18.5–24.9 kg/m^2^) Overweight (25–29.9 kg/m^2^) Obese (>30 kg/m^2^)	ICU In-hospital morality	The study was not able to validate the obesity survival paradox in COVID-19 infected patients.
8.	Wolf et al. [47]	Retrospective study (*n* = 277) in Boston-USA	RT-PCR	Without obesity (BMI ≤ 29.9 kg/m^2^) Obesity class 1 (30 to 34.9 kg/m^2^) Obesity class 2 (35 to 39.9 kg/m^2^) Obesity class 3 (≥40 kg/m^2^)	ICU admission Survival	The obesity was not significant associated with clinical outcomes, the study not being able to demonstrate an obesity survival paradox in COVID-19 infected patients.
9.	Carneiro RAVD et al. [48]	Retrospective study conducted in Brazil	RT-PCR	Overweight (BMI ≥ 25 kg/m^2^) Obesity (BMI ≥ 30 kg/m^2^)	Mortality	There is a positive corelation between obesity and overall mortality.
10.	Foulkes et al. [49]	Retrospective study conducted in USA (*n* = 3828)	RT-PCR	Obesity (BMI >30 kg/m^2^)	IMV Mortality	Obesity increases the systemic inflammation response COVID-19 patients and leads to severe outcomes.
11.	Motaib et al. [50]	Retrospective study in Morocco (*n* = 107)	RT-PCR	Obesity (BMI ≥ 30 kg/m^2^)	ICU admission	Obesity is independently associated with an increased rate of ICU admission.
12.	Sidhu et al. [51]	Retrospective study (*n* = 425) in New Orleans, USA	RT-PCR	Obesity (BMI >30 kg/m^2^) Severe obesity (≥35 kg/m^2^ and < 40 kg/ m^2^) Morbid obesity (>40.0 kg/m^2^)	Mortality	COVID-19 obese patients with at least one obesity related condition have an increased risk of death.
13.	Yates et al. [52]	Retrospective study (*n* = 412,596) in UK	RT-PCR	Normal weight (18.5 to 24.9 kg/m^2^) Overweight (25.0 to 29.9 kg/m^2^) Obesity (BMI ≥ 30 kg/m^2^)	Mortality	Obesity is associate with a higher rate of Mortality
